# Two to Tango: Co-evolution of Hominid Natural Killer Cell Receptors and MHC

**DOI:** 10.3389/fimmu.2019.00177

**Published:** 2019-02-19

**Authors:** Emily E. Wroblewski, Peter Parham, Lisbeth A. Guethlein

**Affiliations:** ^1^Department of Anthropology, Washington University, St. Louis, MO, United States; ^2^Departments of Structural Biology and Microbiology & Immunology, Stanford University School of Medicine, Stanford, CA, United States

**Keywords:** NK cells, MHC, KIR, CD94, NKG2, hominid, great ape

## Abstract

Natural killer (NK) cells have diverse roles in hominid immunity and reproduction. Modulating these functions are the interactions between major histocompatibility complex (MHC) class I molecules that are ligands for two NK cell surface receptor types. Diverse killer cell immunoglobulin-like receptors (KIR) bind specific motifs encoded within the polymorphic MHC class I cell surface glycoproteins, while, in more conserved interactions, CD94:NKG2A receptors recognize MHC-E with bound peptides derived from MHC class I leader sequences. The hominid lineage presents a choreographed co-evolution of KIR with their MHC class I ligands. *MHC-A, -B*, and *-C* are present in all great apes with species-specific haplotypic variation in gene content. The Bw4 epitope recognized by lineage II KIR is restricted to MHC-B but also present on some gorilla and human MHC-A. Common to great apes, but rare in humans, are MHC-B possessing a C1 epitope recognized by lineage III KIR. *MHC-C* arose from duplication of *MHC-B* and is fixed in all great apes except orangutan, where it exists on approximately 50% of haplotypes and all allotypes are C1-bearing. Recent study showed that gorillas possess yet another intermediate *MHC* organization compared to humans. Like orangutans, but unlike the *Pan-Homo* species, duplication of *MHC-B* occurred. However, *MHC-C* is fixed, and the MHC-C C2 epitope (absent in orangutans) emerges. The evolution of MHC-C drove expansion of its cognate lineage III KIR. Recently, position −21 of the MHC-B leader sequence has been shown to be critical in determining NK cell educational outcome. In humans, methionine (−21M) results in CD94:NKG2A-focused education whereas threonine (−21T) produces KIR-focused education. This is another dynamic position among hominids. Orangutans have exclusively −21M, consistent with their intermediate stage in lineage III KIR-focused evolution. Gorillas have both −21M and −21T, like humans, but they are unequally encoded by their duplicated *B* genes. Chimpanzees have near-fixed −21T, indicative of KIR-focused NK education. Harmonious with this observation, chimpanzee KIR exhibit strong binding and, compared to humans, smaller differences between binding levels of activating and inhibitory KIR. Consistent between these MHC-NK cell receptor systems over the course of hominid evolution is the evolution of polymorphism favoring the more novel and dynamic KIR system.

## Introduction

**Figure 1:**
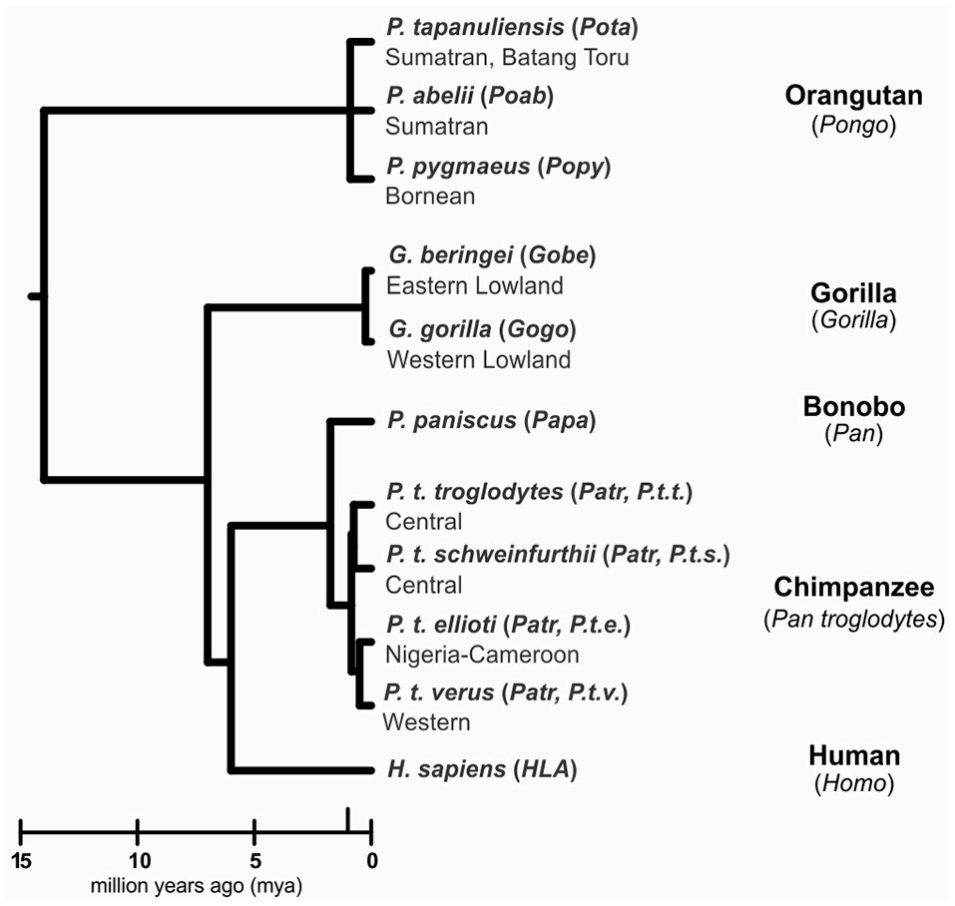
Phylogeny of the great apes. Branch lengths of the tree correspond to divergence time estimates (1, 2). Shown are the scientific name (italics), abbreviation (in parentheses) and common name for the great ape species discussed in this review.

Humans (*Homo sapiens*) are considered a great ape, together with species from three other genera: *Pan* (chimpanzee and bonobo) and *Gorilla* (two species), both of which are African, and *Pongo*, the only Asian genus (orangutan, three species) (1, 2) (Figure 1). All extant and extinct species of these genera are members of the taxonomic family known as hominids (*Hominidae*). Comparative analyses of humans with their closest living relatives have provided insights into the evolutionary origins and interactions between two complementary systems of natural killer (NK) cell receptors and their major histocompatibility complex (MHC) class I ligands. Studies of human, chimpanzee, and orangutan killer cell immunoglobulin-like receptors (KIR) and their MHC class I ligands provided a model for the co-evolution of hominid *KIR* and *MHC* genes (3–8). More recently published studies of gorillas (9, 10) and bonobos (11–13), as well as continued analysis of orangutan (14) have expanded knowledge of MHC class I diversity and polymorphism in these species. Using these new data to expand on the current model, we show how gorillas share features of MHC class I with orangutan, and how targeted gene losses in the bonobo *KIR* locus (4) correlate with changes in the MHC class I repertoire.

The interaction of KIR with cognate MHC class I ligands is an important and diversifying feature of the NK response of humans, apes and Old World monkeys. In all aspects of NK cell biology KIR cooperate with CD94:NKG2A, another HLA class I receptor on NK cell surfaces (15, 16). CD94:NKG2A and KIR have completely different molecular structures (17), but similar functions. Interaction of CD94:NKG2A with its nonclassical MHC class I ligand, HLA-E, is conserved in human populations (18–21). In striking contrast, the interactions of KIR with their classical MHC class I ligands, HLA-A, -B, and -C, are highly variable (22–29). Although mature HLA-A, -B, and -C glycoproteins bind to KIR, a nonamer peptide cleaved from their leader sequences specifically binds to HLA-E, thereby forming the ligand recognized by CD94:NKG2A (30–33). At position −21 of the leader peptide of HLA-B, there is a polymorphism between methionine (M) and threonine (T) maintained in human populations (34). Leader sequences with −21M give a peptide that binds tightly to HLA-E, enabling it to reach the cell surface and be recognized by CD94:NKG2A on NK cells (35, 36). On the contrary, −21T leader sequences give peptides that bind poorly to HLA-E, which is then retained inside the cell and degraded (36).

The immediate consequence of −21M polymorphism of HLA-B is to vary the amount of HLA-E at cell surfaces: the amount being highest for M/M individuals, lowest for T/T individuals and intermediate for M/T individuals (34). These simple differences have a profound influence on the development of NK cells and how they respond to infection and cancer (37–39). During development, the immature NK cells of an individual are educated to recognize the subset of HLA class I isoforms expressed by the individual (40, 41). Playing a crucial role in NK cell education are inhibitory receptors that recognize HLA class I. These receptors are HLA-E specific CD94:NKG2A and the inhibitory KIR that recognize HLA-A, B, and C polymorphisms (40, 41). In people homozygous for −21M HLA-B, NK cell education is dominated by CD94:NKG2A, whereas NK cell education in −21T HLA-B homozygotes is dominated by inhibitory KIR (34). Our initial comparison of the −21M/T dimorphism in apes and humans pointed to species-specific differences (34). With the new data for orangutans (14), gorillas (9), bonobos (11, 12), and chimpanzees (12) we have now performed a broader and deeper search for such differences.

## Interactions Between MHC Class I And NK Cell Receptors Educate NK Cells

NK cells are a diverse population of lymphocytes that contribute both to innate and adaptive immunity against infection, particularly viral infection (42). NK cells also detect and destroy malignant cells (43). In placental reproduction, NK cells perform an essential role in the formation of the placenta (44, 45). In all three scenarios, NK cell responses are governed by balance between intracellular signals delivered by activating and inhibitory receptors (46, 47). NK cells have much in common with T cells, but a key difference is that the structural variation of T cell receptors is largely somatic in origin, whereas NK cell receptor variation is all of germ line origin. A second important difference is seen in the triggers that activate NK cells and T cells. T cell receptors respond to the presence of pathogen- or tumor-derived peptides that are bound by MHC class I and presented on the surface of infected or malignant cells. Conversely, NK cell receptors respond to cells in which the surface level of MHC class I has become abnormally low, or even lost. In this context, NK cells are said to respond to the self-MHC class I that is missing, and in so doing make a “missing-self” response (48). Reduction or loss of MHC class I is a frequent occurrence for virus-infected and tumor cells, because it allows these cells to escape from T cell immunity (49). Countering the escapees are NK cells, which respond to the loss of MHC class I and become activated to kill the tumor or virus-infected cells. Loss of MHC class I is detected by the inhibitory MHC class I receptors that NK cells express (49). These inhibitory receptors prevent NK cells from attacking healthy cells expressing normal levels of MHC class I (50). However, when the NK cell interacts with unhealthy cells compromised in MHC class I expression, their inhibitory signaling diminishes or is lost allowing the NK cell to respond and kill the cells (51). During NK cell development, immature NK cells learn to distinguish between healthy cells expressing normal MHC class I levels and unhealthy cells with dangerously low MHC class I expression (51). As consequence of this learning process, called NK cell education, all functional NK cells acquire an inhibitory CD94:NKG2A or KIR receptor that recognizes one or more self MHC class I isoforms (41).

The molecular mechanisms in the intracellular signaling pathways that underpin NK cell education are incompletely understood but are the subject for much ongoing research (41, 51). Generally agreed upon is that the response of an NK cell is determined by a balance of signals coming from batteries of activating and inhibitory receptors (52). Over the course of NK cell education these signaling pathways become tuned and calibrated (47). Engagement of healthy tissue cells by educated NK cells causes the inhibitory MHC class I receptors to deliver negative signals that overwhelm the positive signals produced by the activating receptors (47, 50, 51). On the other hand, if tissue cells are unhealthy and have reduced amounts of MHC class I on their plasma membranes, then activating signals prevail and the unhealthy cells become targets for NK cell killing (52).

The phenomenon of NK cell education has been principally studied in mice and humans (41). Although human KIR and mouse Ly49 receptors are structurally disparate, the structure and function of their signaling motifs are remarkably similar (46, 53–55). In addition to expression of an inhibitory self-MHC class I receptor, the population of educated NK cells express diverse combinations of receptors and other cell surface proteins that distinguish them from immature NK cells (51). A similar level of phenotypic diversity has been described for human (27, 56, 57), chimpanzee (3, 58), and rhesus macaque (59–63). These observations raise the possibility that humans, apes and Old World monkeys all educate their NK cells in a similar fashion.

## One Conserved and One Polymorphic System of MHC Ligands And NK Cell Receptors

Two MHC class I-interacting NK receptor systems cooperate in non-human primates, the older and more conserved CD94:NKG2 system and the diverse and evolutionarily dynamic KIR. The CD94:NKG2 receptors are heterodimers composed of two lectin-like proteins encoded in the natural killer complex (NKC) (53). The constant partner in the heterodimer is CD94, which in humans can pair with NKG2A, C, or E (31). Of these three, only NKG2A has an inhibitory ITIM motif in its cytoplasmic domain and is thus able to transmit inhibitory signals when engaged (64). In contrast, NKG2C and NKG2E both have charged residues in their transmembrane domain that interact with DAP-12 to transmit activating signals (65, 66). The CD94:NKG2E receptor is retained intracellularly due to hydrophobic amino acids found in its intracellular domain (66). In humans the binding partner for CD94:NKG2A and CD94:NKG2C is the conserved, non-classical MHC class I molecule HLA-E (31, 32). Cell surface expression of HLA-E and its ability to bind CD94:NKG2 is peptide dependent (35, 36, 67). The predominant peptide bound by HLA-E is cleaved from the leader peptide of HLA class I sequences (30, 31). This positions the CD94:NKG2 receptor to provide overall surveillance of HLA class I expression by target cells. There are polymorphisms in the MHC class I leader sequence that either enhance or abrogate peptide binding to HLA-E and subsequent cell surface expression (35, 36, 68). Differences of HLA-E expression due to position −21 leader sequence polymorphism affect the educational programming of NK cells (34). Additional polymorphisms in the leader sequence can also disrupt binding to CD94:NKG2 (68, 69). Orthologs for *HLA-E* have been identified in all primates examined, and phylogenetic analysis indicates it is a product of the oldest of the duplications that formed the primate MHC (70). The *CD94:NKG2* genes are similarly conserved in primate evolution with few variations observed within the apes (20). Modest expansion of *NKG2C* genes occurred in the rhesus macaque (63).

**Figure 2:**
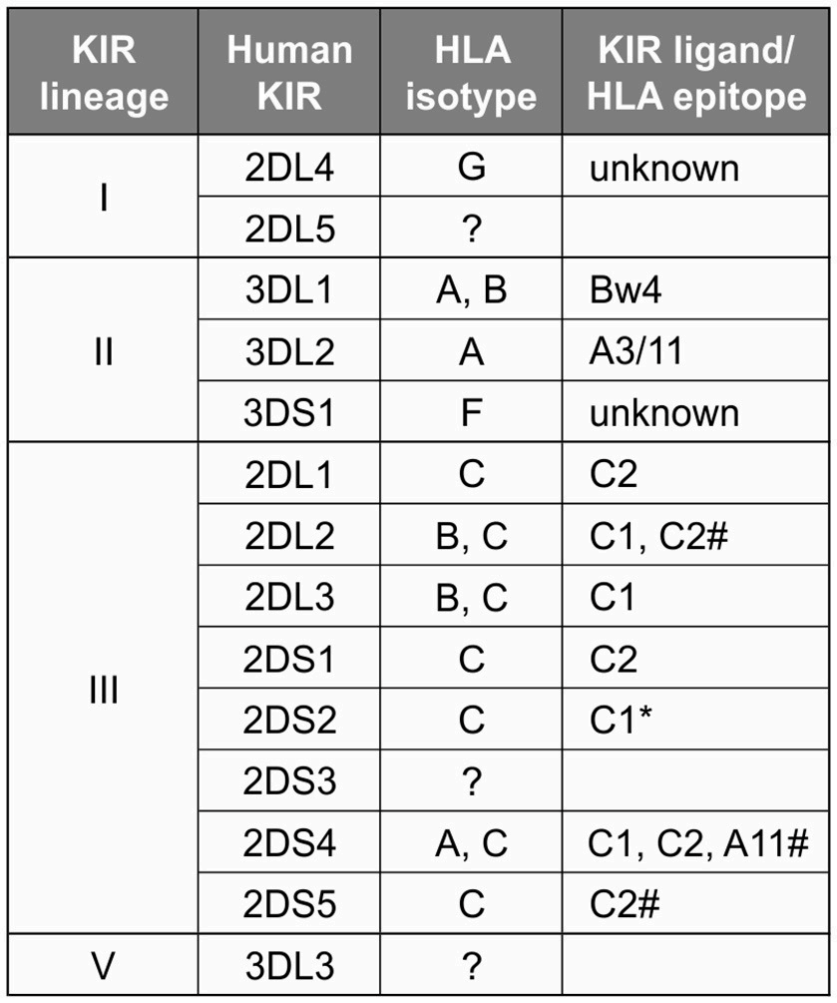
Human KIR lineages, epitopes, and ligands. The four KIR lineages present in great apes are shown on the left with the human members of each lineage given in the second column. There are four haplotype framework genes: *KIR2DL4, KIR3DL2, KIR3DL3*, and a pseudogene, *KIR3DP1* (lineage III, not shown). The third column gives the HLA isotypes having the epitopes recognized by the KIR. Where known, specific binding epitopes are given in the column on the right. Bw4, residues 77–83 with R83 as the key residue recognized by lineage II KIR (74–76); A3/11, unknown residues; C1, V76 K80; C2, N80 (77, 78). ^*^ Binding of 2DS2 to C1 is dependent on binding an appropriate peptide (79). # for these interactions only subsets of molecules with the listed epitope bind (28, 80, 81).

In contrast to the partnership of conserved MHC-E and CD94:NKG2, that of KIR and its classical MHC class I ligands has been changing throughout primate evolution (8). Great ape KIR are transmembrane proteins having either two or three extracellular immunoglobulin-like domains (KIR2D or KIR3D) and either “long” (L) or “short” (S) cytoplasmic tails. KIR with “long” cytoplasmic tails have ITIMs in their cytoplasmic tail that transmit inhibitory signals upon receptor engagement (71). The “short” tail KIR terminate transcription prior to the encoding of the ITIMs (72). The “short” tail KIR also have a charged residue in the transmembrane domain that can complex with DAP-12 and transmit activating signals after receptor engagement (65). Great ape and Old World monkey KIR are grouped into four phylogenetically distinct lineages having distinct structural characteristics and HLA binding partners (epitopes encoded within HLA isotypes) (58, 73) (Figure 2). Only the great apes have orthologs encoding all of the human MHC class I KIR ligands (classical HLA-A, -B, and -C, and nonclassical HLA-F, -G) (6). The human, ape, and Old World monkey *KIR* locus has four haplotype framework genes, one of each of the four lineages [in humans they are *KIR2DL4* (lineage I), *KIR3DL2* (lineage II), *KIR3DL3* (lineage V), and a pseudogene, *KIR3DP1* (lineage III)], around which the remainder of the genes are organized (82). In humans, *KIR* haplotypes can be divided into two distinct groups, *KIR A* and *KIR B* (56, 57, 83). The *KIR A* haplotypes primarily encode inhibitory KIR and have been associated with resistance to infectious disease (84, 85), whereas *KIR B* haplotypes encode additional activating receptors and have been associated with protection from pre-eclampsia and other pregnancy disorders (86–88).

## Emergence of the −21T MHC Class I Leader Sequence in African Apes

**Figure 3:**
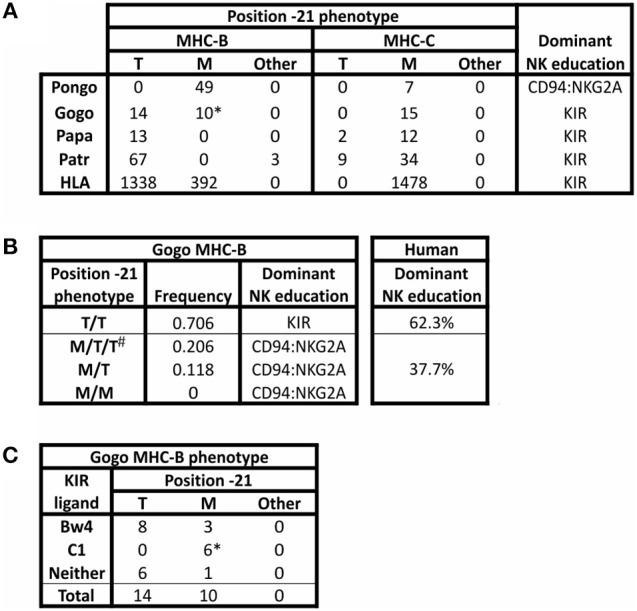
Polymorphism at position −21 of MHC-B and -C. All *MHC-B* and *MHC-C* alleles for which sequence information was available in the IMGT and IPD databases (accessed 9 Aug. 2018) (21, 90) were analyzed for polymorphism at position −21 of the full-length immature protein. **(A)** The number of alleles encoding methionine (M), threonine (T), or other amino acid (Other) is shown for *MHC-B* and *MHC-C*. Isoleucine is the alternative residue for all three Patr-B allotypes under “Other.” All orangutan (Pongo) sequences from *P. abelii* and *P. pygmaeus* have been grouped together; no sequences are available for *P. tapanuliensis*. *Gogo* only includes *G. gorilla* alleles. For *G. beringeri*, the one MHC-B sequence has arginine −21 and the one MHC-C sequence has −21M (not shown). The final column shows the predicted bias in NK cell education based on the relative abundance of −21T (34). **(B)** Phenotypic frequencies for MHC-B position −21 alleles in a panel of 34 Western gorillas (Gogo) (9, 10) are given. Based on the results of Horowitz et al. (34), the predicted dominant mode of NK cell education is indicated. The phenotypic frequencies of human HLA-B position −21 alleles are shown for comparison. The data are from 8,192 individuals representing 51 populations worldwide (34). ^#^Gorillas can have more than two Gogo-B variants because all gorillas have one fixed *Gogo-B* gene, and some gorillas also have an additional related Gogo-B^*^07 gene, which is present on some *Gogo* haplotypes but not on others (9, 10). **(C)** Association of KIR ligands with position −21M polymorphism in Gogo-B are shown. ^*^ Indicates that all four B^*^07 alleles from the Gogo-B^*^07 gene are included in the count.

The threonine/methionine polymorphism at position −21 of the human HLA-B leader sequence corresponds to position 2 of the nonamer peptide bound by HLA-E (31, 89). Binding of this peptide to HLA-E is stronger when methionine is at this position than when threonine (T) is at this position (36). Methionine (M) is at position −21 in almost all HLA-A and HLA-C leader peptides (34) (Figure 3A). Individuals with −21M in five or six of their six expressed classical MHC class I allotypes (HLA-A, -B, and -C) have increased cell surface expression of HLA-E, compared to the dampened HLA-E expression of individuals with −21T in both HLA-B leader peptides (34). Higher expression of HLA-E is further associated with a bias in NK cell education facilitated by the older, more conserved interactions between MHC-E and CD94:NKG2A, whereas NK cell education is facilitated by KIR when there is lower expression of HLA-E. The majority of HLA-B encode −21T compared to −21M (Figure 3A). While there is worldwide population variation, this results in most humans being T/T homozygotes and, therefore, biased toward NK cell education mediated by KIR (Figure 3B).

To determine the ancestral residue at position −21 of MHC-B, Horowitz et al. (34) examined position −21 diversity in the MHC-B sequences of other species of non-human primates. Two lines of evidence support methionine as the ancestral residue at position −21. First, in Old World monkeys, such as the rhesus macaque, methionine is the predominant residue at position −21 in MHC-A and -B (macaques lack MHC-C). Furthermore, all orangutan MHC-A, -B, and -C allotypes have methionine at position −21 (17, 24, and 7 allotypes, respectively) (Figure 3A). Second, analysis of the amino acids encoded at position −21 showed that every alternative to methionine, including −21T, could have been derived by point mutation in the methionine codon, ATG. However, this is not the case when any of the alternative residues to −21M are considered as the ancestral form.

Recent description of gorilla and bonobo MHC class I sequences increases support for methionine having been the ancestral residue at position −21 (9, 10, 12). Methionine is the dominant residue at position −21 in African ape MHC-A and MHC-C (Figure 3A). None of the alternative residues is present at high frequency. For MHC-A, the alternative residues include arginine, encoded by one of the 12 *MHC-A* alleles in Western gorillas (*Gorilla gorilla, Gogo*), isoleucine encoded by only one of the 46 *MHC-A* alleles in chimpanzees (*Pan troglodytes* (*P. t.), Patr*), and leucine encoded by only one of the 24 *MHC-A* alleles in bonobos (*Pan paniscus, Papa*). African ape *MHC-C* alleles all encode −21M, except for −21T encoded by two of 14 bonobo alleles and nine of 43 chimpanzee alleles. For MHC-B, the alternative form, −21T, is present in all species of African apes at varying frequencies (Figure 3A). However, −21T is not found in Asian ape MHC-B or -C, suggesting −21T emerged after the divergence of African and Asian apes (34), ~13–14 mya (1).

**Figure 4:**
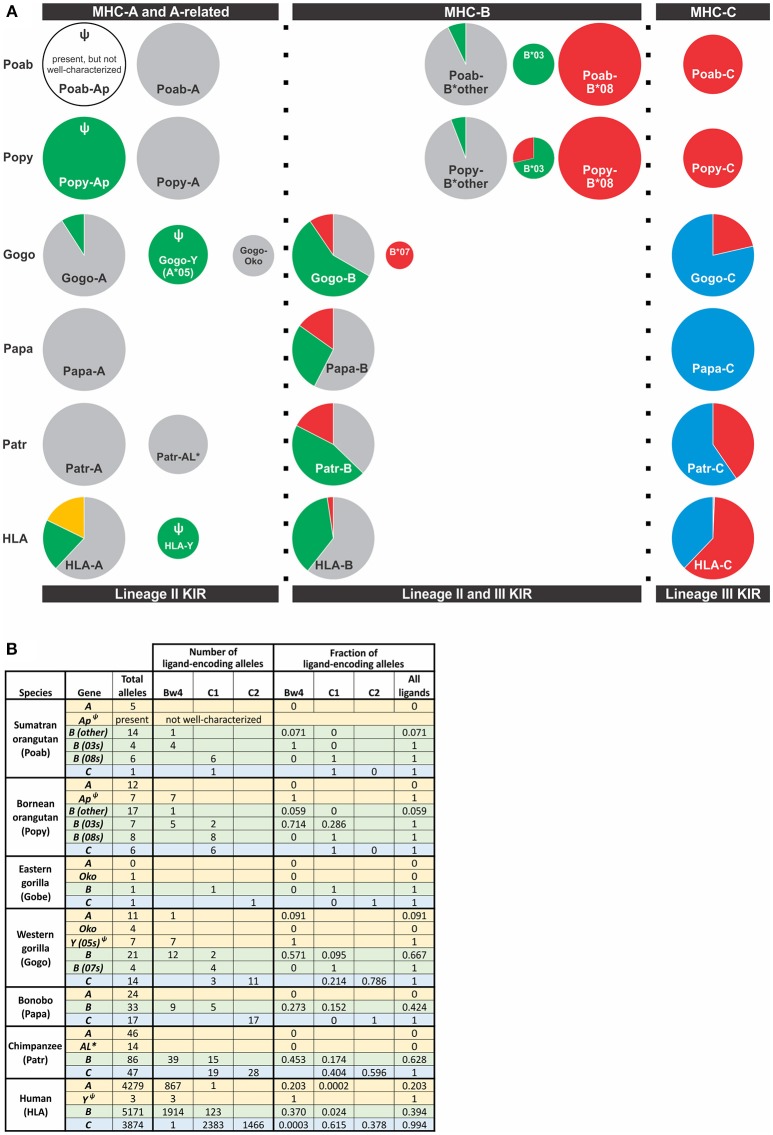
Distribution of alleles encoding a KIR ligand among great ape *MHC class I* genes. Alleles included were those for which sequence information was available in the IMGT and IPD databases (Accessed Aug. 9, 2018) (21, 90). **(A)** The frequency of alleles encoding a KIR ligand for *MHC-A, -A-*related, *-B*, and *-C* genes is shown. Each circle represents a gene and is scaled so that the area of the circle is proportional to the frequency of the gene among *MHC* haplotypes. Orthologous genes are aligned vertically. KIR ligands are color-coded; gold, A3/11; Bw4, green; C1, red; C2, blue; no ligand, gray. Ψ indicates a pseudogene. *Indicates a non-classical gene. Two Popy-B*03 encode a Bw4-C1 hybrid epitope that are categorized as C1. **(B)** The table provides the numbers and frequencies used for the pie charts in panel **(A)**.

Comparison of twenty-four Western gorilla *Gogo-B* alleles (9, 10) showed a balanced polymorphism in which 14 encode −21T and 10 encode −21M (Figure 3A). Included in this analysis are alleles of the fixed *Gogo-B* gene, as well as from a related gene that is not fixed (9). This second gene, which is defined by the four Gogo-B^*^07 alleles, was present in only eight of 35 gorillas (22.8%) examined (9) (Figure 4). Phylogenetic analysis showed that the Gogo-B^*^07 gene is more closely related to orangutan *MHC-B* than to *Gogo-B* (9). Like those of orangutan MHC-B, Gogo-B^*^07 alleles all encode −21M (Figure 3A). In contrast, the phylogenetic analysis showed that alleles of the fixed *Gogo-B* gene are more closely related to *MHC-B* of other African apes than to orangutan *MHC-B* or Gogo-B^*^07 (9). Polymorphism at position −21 is only observed in the 20 alleles of the fixed *Gogo-B* gene and is biased toward −21T: −21T (14 alleles) and −21M (6 alleles). The shared occurrence of −21M encoded within both gorilla *MHC-B* genes is further support of −21M as the ancestral and −21T as the derived amino acid, its emergence occurring specifically within the ancestor of the fixed and more African-like *Gogo-B* gene. Furthermore, the combined effect of −21T skew within the fixed *Gogo-B* and the low frequency of Gogo-B^*^07 among gorillas results in 70% of gorillas being phenotypically homozygous for −21T (Figure 3B), despite the approximate balance of −21M and −21T encoded among all 24 *Gogo-B* and Gogo-B^*^07 alleles. Most Western gorillas, therefore, are predicted to favor NK cell education mediated via MHC class I and KIR interactions, and in proportions similar to those observed in human populations (34) (Figure 3B).

Unlike the −21 polymorphism in human and gorilla MHC-B, both *Pan* species, chimpanzee and bonobo MHC-B are dominated by −21T (Figure 3A). All bonobo *Papa-B* alleles and all but three chimpanzee *Patr-B* alleles encode −21T. The exceptions are the three older and divergent Patr-B^*^17 alleles (91). Ancestral *Pan* is believed to have experienced a selective sweep ~2–3 mya, before the two species lineages diverged (Figure 1), causing the loss of the *A2* lineage of *MHC-A* alleles and a general loss of *MHC class I* intronic diversity (12, 91–93). The reduction of intronic variation was more severe for *MHC-B* of chimpanzees and bonobos than for *MHC-A* or *-C* (11, 12, 93), strongly suggesting that a selective sweep acted specifically on *MHC-B*. This selective sweep in ancestral *Pan* could account for the loss of −21M and near fixation of −21T encoded by *MHC-B*. The consequence of the selective sweep is a system in *Pan* that is strongly biased toward NK cell education mediated by MHC class I and KIR interactions.

A candidate cause of the selective sweep in *Pan* is a lentivirus related to SIV and HIV. Nearly all African primates are infected with a species-specific SIV virus, including the colobus and other monkeys upon which chimpanzees and bonobos prey (94–96). Among the great apes, Western gorillas are known to be infected with SIVgor in the wild (97). Although SIV infection is absent in extant bonobos (98), SIVcpz is naturally occurring in extant chimpanzees, however only within the central *P. t. troglodytes* and eastern *P. t. schweinfurthii* chimpanzee subspecies (94, 98). This suggests that extant chimpanzee SIVcpz infection originated recently, after the divergence of the *P. t. troglodytes* and *P. t. schweinfurthii* subspecies from the western *P. t. verus* and Nigeria-Cameroon *P. t. ellioti* subspecies (Figure 1). Viral phylogeny points to SIVcpz being evolutionarily young, having existed within chimpanzees for approximately 500 years (99). Nonetheless, related lentiviruses have likely plagued African primates for millions of years (94) and been a sustained selection pressure during *Pan* evolution (94, 100). In further support of a lentiviral selective sweep driving the near fixation of −21T in *Pan*, the −21 T/T HLA-B phenotype in humans is associated with NK cells that are more effective in resisting HIV infection, as well as the disease progression to AIDS, compared to NK cells in individuals with the M/M or M/T phenotype (34, 37, 101, 102). In addition, a conserved clade of *MHC-B* alleles that is present in humans, chimpanzees, and gorillas and is associated with protective effects against the progression of HIV and SIV infection (92, 100, 103) also has a predominance of −21T MHC-B allotypes. The clade includes human HLA-B^*^57:01, which is associated with long-term non-progression to AIDS (104–109) and encodes −21T. The clade also includes many chimpanzee *Patr-B* alleles, most of which (95.7% of *Patr-B*) have −21T (Figure 3A). Also in the clade are gorilla alleles, Gogo-B^*^03:01 and ^*^04:01, which encode −21T. This unusually conserved clade further associates −21T with protective effects against lentiviruses. Intriguing is that new MHC-C alleles were recently identified in chimpanzees and bonobos (12) which encode −21T (Figure 3A). While not frequent among the animals genotyped (12), these variants further suggest that selection has favored −21T and KIR-biased NK cell education and immune response.

Coexisting with the −21 polymorphism of classical MHC class I are encoded epitopes that serve as ligands for KIR. The current dataset of gorilla Gogo-B reveals a bias in the occurrence of −21T within in the subset of allotypes encoding the Bw4 epitope that is recognized by lineage II KIR (Figure 3C). Eight of 11 Bw4-encoding alleles have −21T (Figure 3C). This skewed distribution is consistent with the emergence of −21T in an allele encoding the Bw4 epitope. This would have created an association between an allotype having a KIR ligand and a leader peptide sequence that would have dampened NK cell education through MHC-E and CD94:NKG2A. Together these effects would promote education mediated by KIR. Gorillas also have the C1 epitope that is encoded by six of the 24 *Gogo-B* alleles and is recognized by lineage III KIR. This is a greater proportion of C1-encoding *MHC-B* than in humans, in which C1^+^HLA-B has been largely eliminated (except for HLA-B^*^73 and HLA-B^*^46) (110) (Figure 4). The C1 epitope of gorilla Gogo-B is in complete association with −21M. However, the majority of these C1-encoding *Gogo-B* alleles (four of six) are alleles of the duplicated Gogo-B^*^07 gene that is both unfixed and infrequent (9) (Figure 3C). The association between the C1 epitope and −21M of the unfixed and more Asian-like Gogo-B^*^07 gene (9) is therefore an older form of MHC-B and is also found in orangutan MHC-B. The near-fixation of −21T within the single MHC-B of chimpanzee and bonobo (Figure 3A) means the encoded Bw4 and C1 epitopes are in complete association with −21T. This association promotes strong NK cell education by KIR in chimpanzees. In addition, all *MHC-C* alleles encode epitopes that are lineage III KIR ligands (either C1 or C2) (Figure 4). These MHC-C KIR ligands are nearly always found in association with −21M. However, a recently discovered subset of chimpanzee and bonobo *MHC-C* alleles encodes −21T (Figure 3A). These alleles are predicted to further the bias in these species toward NK cell education by KIR. Humans have retained the preferential association of Bw4^+^HLA-B with −21T [only HLA-B^*^38, an allotype that may have originated in archaic *Homo* (110), has Bw4 associated with −21M (34)]. However, −21M has reemerged among human HLA-B and is found primarily on alleles lacking ligands recognized by KIR (34), suggesting there has been some selection for NK education mediated by MHC-E and CD94:NKG2A in the human lineage.

All human populations have HLA-B allotypes with methionine and threonine at position −21. While their relative frequencies vary between populations, the majority of populations (and the individuals within them) exhibit a dominance of −21T (34). Thus, humans, like for gorillas, chimpanzees, and bonobos, are heavily biased toward NK cell education via MHC class I and KIR interactions (Figure 3B). Interestingly, −21M is found at highest frequency among Europeans but at intermediate frequency among African and Asian populations (34). This suggests that Europeans have experienced local selection in favor of −21M and NK cell education mediated via interactions between MHC-E and CD94:NKG2A. This regional pattern also suggests the frequency of −21M may have been bolstered by admixture with and adaptive introgression of −21M HLA-B^*^07:02 from archaic humans, among other possible alleles (34, 110).

## Polymorphic Class I *MHC-C* Emerges and Lineage III *KIR* Expand in Orangutans Alongside *MHC-B* Duplication

Characteristic of Old World monkeys, the best studied being the rhesus macaque (*Macaca mulatta*), is a dominance of classical MHC class I-lineage II KIR interactions. Lacking an *MHC-C* gene, KIR interactions in the macaque occurs entirely by epitopes encoded by *MHC-A* and *MHC-B*, both of which can be duplicated one or more times on the same haplotype (111–113). Rhesus macaques have a corresponding breadth of lineage II *KIR* genes, with 19 genes identified and extensive haplotypic diversity (ranging between four and 15 lineage II *KIR*) (114–118). Hominoid species, including lesser and great apes, have taken different paths than the Old World monkeys. Of the species examined, lesser apes (gibbons) combine a single *MHC-A* gene with multiple *MHC-B* genes (119). Rather than having a large number of lineage II *KIR*, however gibbons have a contracted *KIR* locus giving rise to haplotypes that have only two to five functional *KIR* genes, with lineage V *KIR3DL3* as the only gene present on all gibbon *KIR* haplotypes. Uncertain is the extent to which gibbon NK cells are educated by the interactions of KIR and their MHC class I ligands.

Hominoids further diverged with the emergence of *MHC-C* among the great ape lineage (Figure 4). Orangutans, of which there are three species identified [*Pongo pygmaeus* (Bornean, *Popy*), *Pongo abelii* (Sumatran*, Poab*) (120), and the recently-described *Pongo tapanuliensis* (Sumatran, *Pota*) (2)], represent an evolutionary intermediate on the pathway of great ape classical *MHC class I* and *KIR* coevolution. Orangutans are the earliest-diverged of the extant ape species that also have all three of the classical *MHC class* I genes, *-A, -B*, and *-C*, present in humans (3, 121–123), indicating that *MHC-C* originated prior to the divergence of Asian and African great apes ~13–14 mya (Figure 1) (1, 121). Of the two species best studied, *P. pygmaeus* and *P. abelii*, both possess *MHC-C*, however, the *MHC-C* gene occurs within a dynamic region of the *MHC* locus and is not fixed like the *MHC-C* of humans and African great apes (14, 121). *MHC-C* is present on approximately half of the orangutan *MHC* haplotypes, situating orangutans as an evolutionary intermediate along the path of *MHC-C* evolution. The orangutan *MHC-B* genes vary in number (Figure 4), as in Old World monkeys and gibbons, with two to four genes being present on each chromosome (14, 121). In contrast to Old World monkeys, orangutans have one functional *MHC-A* gene (14), as is the case for gibbons (119).

Orangutan MHC -A, -B, and -C differ from HLA-A, -B, and -C in the epitopes they carry and their interactions with KIR. Both Poab-A and Popy-A lack sequence motifs corresponding to the A3/11 and Bw4 epitopes of HLA-A (6) (Figures 2, 4). While all alleles of the *A-*related gene, *Popy-Ap*, encode the Bw4 epitope, *Popy-Ap* is a non-functional pseudogene (124). Orangutan MHC-A is highly transcribed (14), indicating that MHC-A plays an important role in orangutan immunity but, in lacking ligands for KIR, is dedicated to antigen presentation and CD8 T cell mediated immunity. There is, however, a subset of orangutan MHC-B that encode the Bw4 and C1 epitopes (6) (Figure 4). Two orangutan *MHC-B* allelic lineages, MHC-B^*^08 and MHC-B^*^03, represent two different *MHC-B* genes (14) that both encode KIR ligands. MHC-B^*^08 is fixed on *MHC* haplotypes, and all its alleles encode the C1 epitope. In contrast, the B^*^03 gene is only present on a subset of *MHC* haplotypes. The two Poab-B^*^03 allotypes have a Bw4 motif as do four of the six Popy-B^*^03 allotypes. The other two Popy-B^*^03 allotypes have a hybrid epitope comprising elements of both the Bw4 and C1 motifs (and they are categorized as C1-encoding alleles in Figure 4). Of the other orangutan *MHC-B* alleles only one of the 17 *Popy-B* alleles and one of 14 *Poab-B* alleles encode a Bw4 epitope (Figure 4). Thus, among the different orangutan MHC-B, there is a strong division of epitopes recognized by KIR.

Variability in *MHC-B* copy number, as well as allelic polymorphism, indicate that orangutan MHC-B allotypes have a range of potential to educate NK cells through their interactions with KIR. All orangutans have potential for their lineage III KIR to mediate NK cell education and immune response via the C1 epitope of B^*^08 allotypes, but only a subset of individuals also have the potential for mediation via the Bw4 epitope of MHC-B and lineage II KIR (Figure 4). This variation in capacity is compounded by the considerable difference in transcriptional level observed for these genes. MHC-B^*^03 and MHC-B^*^08 are expressed at lower levels than other orangutan *MHC-B*, with MHC-B^*^03 being lower than MHC-B^*^08 (14).

**Figure 5:**
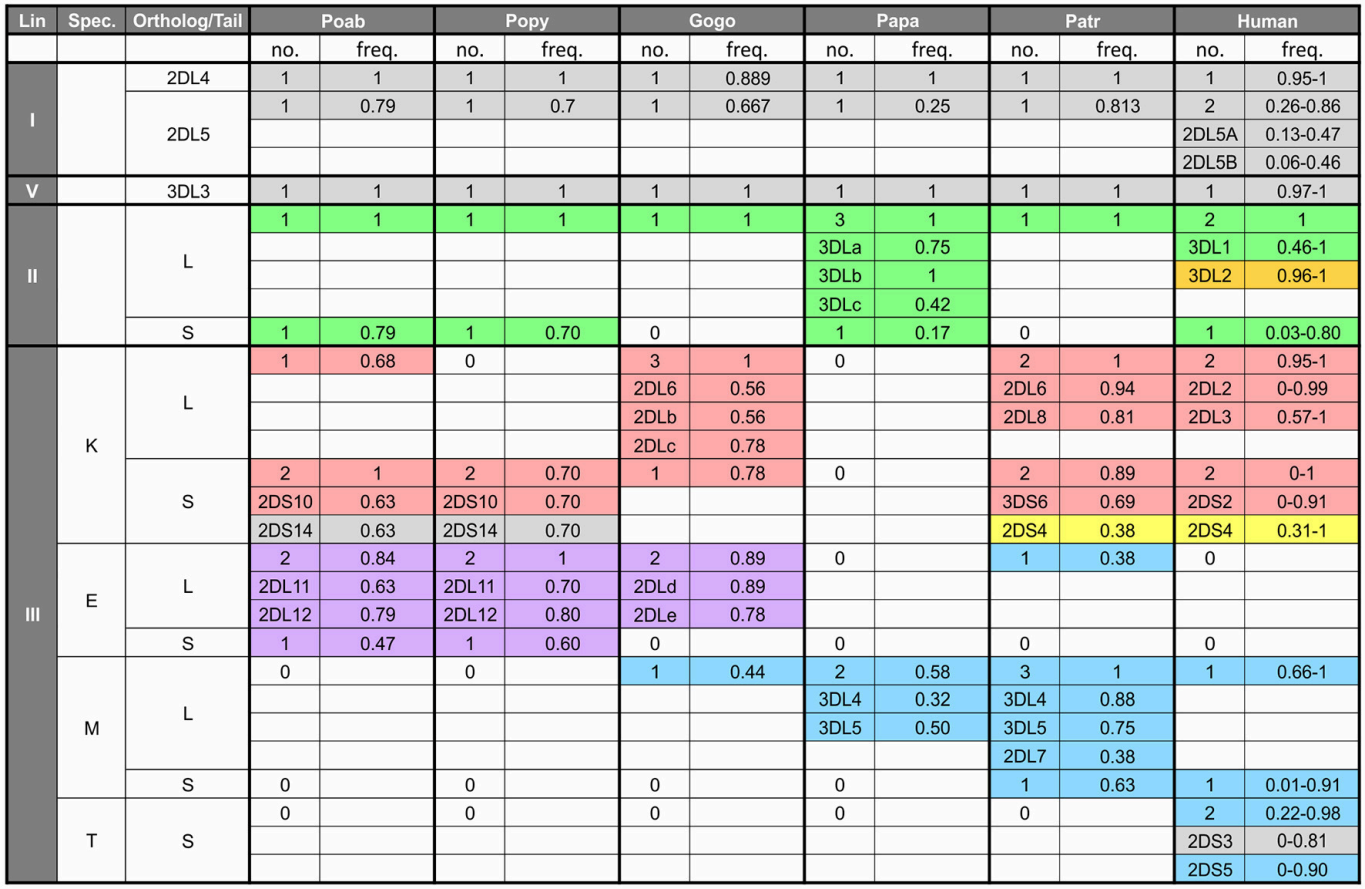
Number and frequency of KIR in great apes. The table shows the number of *KIR* genes in each species according to lineage (first column), specificity-determining residue (position 44) of the lineage III KIR (second column) and type of tail, short (S) or long (L) (third column). For each species the number of genes with the characteristics shown in the first three columns is shown along with the combined *genotypic* frequency. For example, in the cell under *Patr*, and for the characteristics of lineage III—K—S, there are two *KIR* genes (*3DS6* and *2DS4*). The frequency of 0.89 represents the combined frequency of individuals with only *3DS6* (and not *2DS4*), those only with *2DS4* (and not *3DS6*), and those with both genes. In addition, for cells such as this, where there are several genes with a particular set of characteristics, the genotypic frequency for the individual genes are shown—e.g., for *3DS6*, 0.69 is the combined frequency of those individuals with only *3DS6* and those with both *3DS6* and *2DS4*. The data for non-human apes come from the study of captive animals (3, 4, 7, 125), and the values for humans represent the range of genotypic values obtained from the website, allelefrequencies.net (126). “0” indicates absence of a gene encoding the characteristics in the first three columns. Colored shading indicates binding specificity as follows: green, Bw4; red, C1; blue, C2; purple, pan C1/C2; orange, A3/11; yellow, complex pattern of 2DS4; gray, unknown specificity.

All orangutan *MHC-C* alleles encode the C1 epitope (5, 6, 14) (Figure 4), suggesting that orangutan MHC-C is a universal NK cell-educating molecule like MHC-B^*^03 and B^*^08. The *MHC-C* gene is hypothesized to have evolved from the duplication of a C1-encoding *MHC-B*, and this led to all MHC-C allotypes functioning as ligands for lineage III KIR (6). The emergence of *MHC-C* drove the expansion of lineage III *KIR* genes in the centromeric region of the orangutan *KIR* locus (7, 73). Of the seven genes identified, orangutan haplotypes have between one and four centromeric lineage III *KIR* genes (7). The orangutan *KIR* locus has two lineage II *KIR* genes that are predicted to recognize the Bw4 epitope (Figure 5). These are *Popy-* and *Poab-KIR3DL1*, a conserved framework gene, and *Popy-* and *Poab-KIR3DS1*, which is present on a subset of *KIR* haplotypes (7). By contrast, most of the orangutan lineage III KIR are predicted to recognize the C1 epitope which is encoded by some *MHC-B* and all *MHC-C* alleles. Orangutan *MHC-C* is expressed at a low level (14), as is human *HLA-C*, and is comparable to that of orangutan MHC-B^*^03. At position 44 in the ligand binding site orangutan lineage III KIR have either lysine (K44) or glutamic acid (E44) (5). K44 KIR are strong binders and C1-specific, whereas E44 KIR bind less strongly but to both the C1 and C2 epitopes. This dual specificity of E44 lineage III KIR is likely to have facilitated evolution of the human C2 epitope and the C1/C2 functional dimorphism (5, 8).

Recent comparison of *KIR* in *P. pygmaeus* and *P. abelii* shows that substantial changes in have occurred during the time since their divergence (73). The divergence time is 0.3–0.5 million years when estimated from autosomal DNA (1, 120) (Figure 1) but ~3.5 million years when estimated by mitochondrial DNA (120). This discrepancy is consistent with a split of ancestral orangutan populations ~3.5 mya and subsequent male migration between the populations (120). Of the 12 orangutan *KIR* haplotypes identified, only two are shared between the two species. While all *P. pygmaeus* and *P. abelii KIR* haplotypes encode inhibitory C1-specific KIR, only some haplotypes encode activating C1-specific KIR (Figure 5). In an additional functional divergence, Sumatran *P. abelii* haplotypes are enriched for haplotypes that lack genes encoding activating C1 KIR (7). Whereas, five of the seven of the lineage III *KIR* genes are shared by the two species, most of the alleles are species-specific. Thus, while broadly similar in their expansion of lineage III *KIR*, orangutans illustrate how relatively recently evolved species within the same genus can have quite distinctive coevolutionary outcomes of classical MHC class I-KIR interactions for NK cell education and immune response.

## Refinement and Specialization of MHC Class I and KIR in Gorillas

While orangutans are a story of expansion within the *KIR* locus, gorillas are a study of its refinement, one that situates gorillas as another intermediate along the pathway toward human *MHC* and *KIR* organization and function. Of the two species, Western gorillas (*Gorilla gorilla, Gogo*) are better characterized than Eastern gorillas (*Gorilla beringei, Gobe*) and will be the focus here. Superficially, gorilla *MHC-A* and related genes appear similar to orangutan *MHC-A* and *-Ap*: both have a single fixed, polymorphic gene, largely absent of ligands for KIR, and an additional pseudogene (Figure 4). However, phylogenetic analysis points to gorilla *MHC-A* having a more complex history. Polymorphic *Gogo-A* is more similar to the orangutan *A*-related pseudogene, *Popy-Ap*, and the *MHC-A* of other African apes than to orangutan *MHC-A* (9, 124, 127), whereas two additional *A*-related genes, *Gogo-Oko* and *Gogo-Y*, are related to *Popy-A* (9, 128, 129) (Figure 4A)*. Gogo-Y* was previously considered a divergent allele of *Gogo-A* (Gogo-A^*^05) but was recently determined to be a separate gene (9, 128, 130, 131). However, neither *A*-related gene is fixed on gorilla haplotypes (9, 10, 127). Recent analysis of 34 captive animals found *Gogo-Oko* in 44% and *Gogo-Y* in 79% of gorillas (9, 10). Expression patterns suggest Gogo-Oko is a classical antigen-presenting molecule (132). In two of the 34 animals (6%) studied recently *Oko* was the only *A*-related gene present, suggesting it offers sufficient function in the absence of other *Gogo-A* (9). However, the intermediate frequency of *Oko* suggests it could be subject to balancing selection (9). By contrast, while more frequent among gorillas, *Gogo-Y* is mostly pseudogenized, with Gogo-A^*^05:01 remaining as the only functional allele. Gogo-A^*^05:01 was not found in any of the individuals in the study of Hans et al. (9) and has only been observed in the individual from which it was first sequenced (128). The haplotypic variability and functional restriction of the orthologs of the classical orangutan *MHC-A* in gorillas is further observed in chimpanzees and humans. In chimpanzees, the ortholog became a nonclassical gene with low cell-surface expression, *Patr-AL* (124, 133), and a pseudogene in humans, *HLA-Y* (130, 131, 134), neither of which are fixed genes.

**Figure 6:**
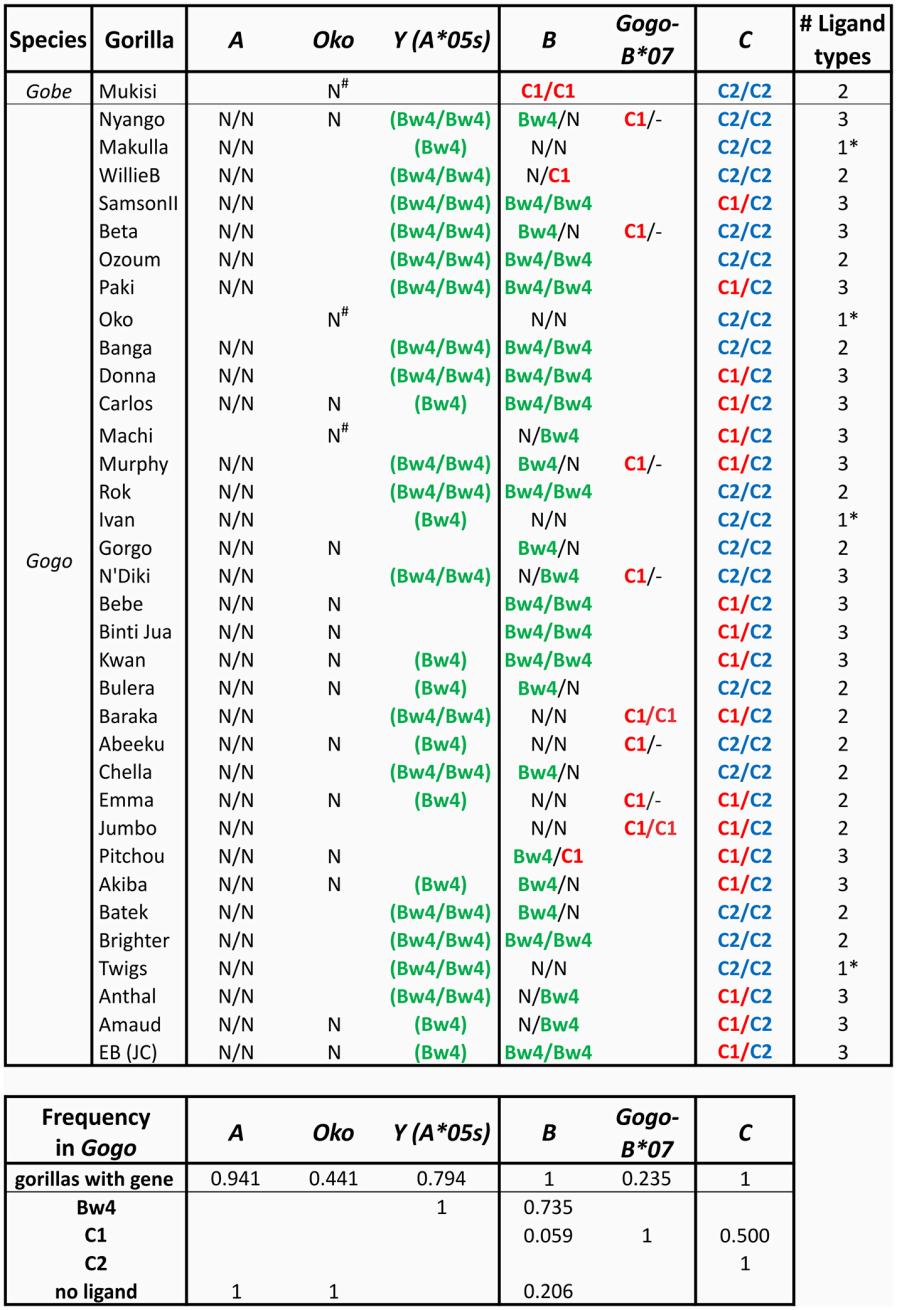
Gorilla MHC class I isotypes and their KIR-binding epitopes. Summarizes data from a panel of 35 gorillas (9, 10). The first column gives the species, the second gives the individual's name. In each of columns 3–8 the presence of a gene and its encoded KIR ligand are given. Presence of a single entry shows the individual lacked the gene on one haplotype, and no entry indicates absence of the gene. Ligands are color-coded as in Figure 3A: Bw4, green; C1, red; C2, blue. N, no ligand; -, absence of Gogo-B*07 (presence on one or both chromosomes of Gogo-B*07 was determined based on patterns of linkage disequilibrium with Gogo-B*03 alleles); parentheses surrounding the ligand indicate that the sequence encoding the epitope is present but in the context of a non-functional (null) allele of *Gogo-Y* (9). The final column gives the number of ligand types present in each individual; *indicates that only one type of ligand is present. The lower panel gives the *MHC* gene frequency and the phenotypic frequency of their encoded ligands.

Gogo-A, -Oko, and -Y have limited capacity to serve as ligands for lineage II KIR (6) (Figure 4). The alleles of *Gogo-Y*, which all encode the Bw4 epitope recognized by lineage II KIR, are largely non-nonfunctional, reminiscent of the alleles of *Popy-Ap*. None of the *Gogo-Oko* alleles encode an epitope recognized by KIR. Just one of 11 *Gogo-A* alleles encode a Bw4 epitope (Gogo-A^*^06:01). Emphasizing the point, none of the 34 captive gorillas recently characterized (9, 10) possess Bw4-bearing MHC-A (Figure 6). Furthermore, none of those animals possess the one functional, Bw4-encoding *Gogo-Y* allele (Gogo-A^*^05:01). However, 57% of *Gogo-B* alleles encode Bw4 (Figure 4), and 74% of the panel of 34 captive gorillas have Bw4-encoding *Gogo-B* (Figure 6). Consequently, lineage II KIR-mediated education and immune response of NK cells via Bw4 are preserved in and driven by Gogo-B, while Gogo-A function appears focused on antigen presentation to T cells. This division of function is also seen in the MHC-A and MHC-B of chimpanzees and bonobos (Figure 4).

African apes were previously considered to all have one *MHC-B* gene, like humans (6, 91, 128, 129, 132, 135, 136). However, recent in-depth characterization of gorilla *MHC class I* identified a second *Gogo-B* gene (9) (Figures 4, 6). We can now conclude that reduction of *MHC-B* to a single gene did not occur in the common ancestor of African apes, as previously thought, but later, after the *Gorilla* lineage diverged from the common ancestor of *Pan* and *Homo* ~7 mya (Figure 1). The second *Gogo-B* gene (Gogo-B^*^07) is not fixed and is present in eight of 34 (24%) animals examined (Figure 6). Gogo-B^*^07 has a recombinant structure that distinguishes it from other *Gogo-B* alleles and which likely gives it functional novelty (9). The frequency of gorillas that have Gogo-B^*^07 is considerably less than those that have *Gogo-Oko* (44%) (9), or chimpanzees that have the nonclassical chimpanzee *Patr-AL* gene (approximately 75%) (124). Unlike *Gogo-Oko*, which can be the sole *MHC-A* gene, Gogo-B^*^07 is never found as the sole *Gogo-B* gene (Figure 6). Intriguingly, Gogo-B^*^07 is more related to orangutan *MHC-B*, whereas the fixed *Gogo-B* gene is most closely related to human and chimpanzee *MHC-B* (9). These characteristics of gorilla *MHC-A* and *MHC-B* suggests that the orangutan-like contributions are specifically the target of reduction, or even elimination, and functional refinement in gorillas and other African apes. By contrast, the *MHC-C* gene, that emerged in the common ancestor of the great apes and is variably present among extant orangutans, became fixed within gorillas and other African apes. The fixation of *MHC-C* in the African apes suggests positive selection on this molecule in their common ancestor, after their divergence from the Asian lineage.

In gorillas NK cell education and immune responses are likely to involve the interaction of lineage II KIR with Bw4^+^MHC-B allotypes. In addition, the presence of the C1 epitope carried by some Gogo-B and -C allotypes indicates that NK cell education is also achieved by the interaction of C1 with lineage III KIR (6, 10) (Figures 4, 6). Of the two gorilla *MHC-B* genes, only alleles of the fixed *Gogo-B* gene encode Bw4 (57%). Only two of 21 (9.5%) *Gogo-B* alleles encode C1. However, the C1 epitope is encoded by all four alleles of Gogo-B^*^07, which is not fixed (Figure 4B). This skewed distribution of KIR ligands parallels that of the orangutan MHC-B^*^03 and ^*^08 genes. Newly emerged among gorilla MHC-C is the C2 ligand for lineage III KIR (6). The C2 epitope is distinguished from the ancestral C1 form by a lysine at position 80 of the peptide-binding domain rather than an asparagine, a switch that only required a point mutation. The presence of C2 among all the African apes is consistent with its emergence after the African ape lineage diverged from that of Asian apes ~13–14 mya (Figure 1).

**Figure 7:**
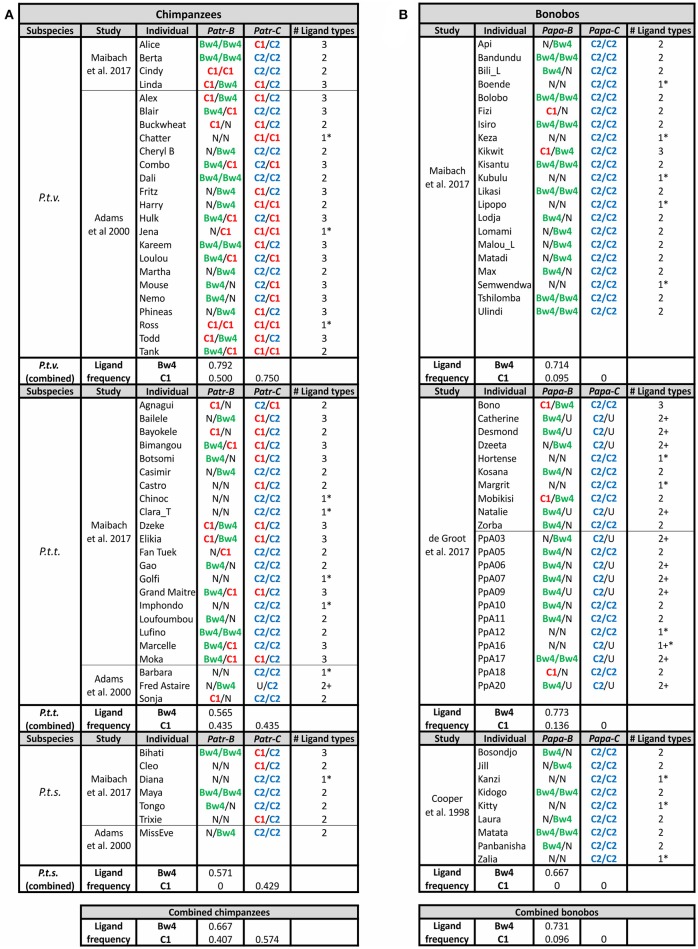
Chimpanzee and Bonobo MHC class I isotypes and their KIR-binding epitopes. Summarizes the *MHC* genotype data obtained in several studies (11, 12, 91, 137). Each study population is shown separately, and the summary of frequencies is given below. Chimpanzees are shown on the left **(A)** and bonobos on the right **(B)**. The color-coding of the KIR ligands is the same as in Figures 3A, 6: Bw4, green; C1, red; C2, blue. U, uncertainty in homozygosity; Phenotypic frequencies are given. * Indicates individuals having a single type of KIR ligand. + Indicates that additional ligands may be present, because of uncertainty in the genotype.

Gorillas that have the C1 epitope via their *MHC-B* genes are relatively rare. In the panel of 34 captive animals (9, 10), only two (~6%) have C1 encoded by the fixed *MHC-B* gene, and eight animals (24%) have a second *MHC-B* gene that encodes C1. No *MHC* haplotype has C1 encoded by both *MHC-B* genes (Figure 6). Thus, 29% of gorillas (10 of 34) have C1^+^MHC-B, while half (50%) of gorillas have C1^+^MHC-C (but they are all heterozygous C1/C2). By contrast, all gorillas have C2^+^MHC-C. In gorillas, four of 34 animals (12%) only have MHC-C acting as a ligand for KIR, and they all were C2 homozygotes (i.e., they are minimally diverse, with just one of the three possible KIR ligand types (Bw4, C1, or C2) represented). While rare in this gorilla panel, this frequency is similar to frequencies observed in human populations, where individuals that lack HLA-A or HLA-B interactions with KIR range from non-existent to near 75% of individuals (8). Individuals with just one ligand type available solely via MHC-C are also observed in chimpanzees and bonobos (Figure 7). Thus, individuals can survive with only these minimal classical MHC class I-KIR interactions directing NK cell activity.

Rather than the two lineage II *KIR* genes present in orangutans, gorillas only have the *3DL1* framework gene (125) (Figure 5). This loss of KIR correlates with the reduction of gorilla *MHC-B* genes to one gene that is fixed and one that is not. While the interactions between Bw4 and lineage II KIR were reduced, an expanded set of seven centromeric lineage III *KIR* genes, six of which are inhibitory, became further specialized with *MHC-C* fixation on haplotypes and the emergence of the C2^+^MHC-C ligand. As with orangutans, gorillas have lineage III KIR that have both K44, conferring C1 specificity (5, 80), and E44, which is associated with pan-C specificity (recognition of both C1 and C2) in orangutans (5). Newly emerging in gorillas, along with the C2 ligand, is a likely C2-specific KIR, Gogo-KIR3DL7, which has the M44 known to have C2 specificity in humans and chimpanzees (138, 139).

## Expansion of Lineage III KIR in Chimpanzees

The common ancestor of *Pan* diverged from that of humans ~6–7 mya (1) (Figure 1). The two extant *Pan* species, chimpanzees (*Pan troglodytes*) and bonobos (*Pan paniscus*), share with humans the same organization of classical *MHC class I* genes. After divergence of the common ancestor of *Pan* and *Homo* from the gorilla lineage, the classical *MHC class I* genes were reduced in number to give single *MHC-A, -B*, and *-C* genes (Figure 4). In *Pan*, as in orangutan, MHC-A appears dedicated to antigen presentation and T cell-mediated adaptive immunity. Neither *Pan* species has *MHC-A* alleles encoding epitopes known to be recognized by human KIR, compared to the small subset of gorilla *Gogo-A* (9.1%) and larger subset of human *HLA-A* alleles (38%) that encode KIR ligands (6) (Figure 4). However, the Bw4 ligand is preserved by a subset of MHC-B allotypes in chimpanzee (45.3%) and bonobo (27.3%) (6) (Figure 4B). Compared to Bw4, the C1 epitope is carried by a smaller subset of *MHC-B* alleles in both species (17.4% of *Patr-B* and 15.2% of *Papa-B*). Like the MHC-C of all the great apes, all *Pan* MHC-C carry a ligand for KIR (6) (Figure 4), either the more ancestral C1 ligand or the C2 ligand that arose among the African ape lineage. Despite their similarities in MHC, the two *Pan* species have marked differences in their KIR.

Chimpanzees have a single lineage II *KIR* gene which shares some components with human lineage II *KIR3DL1* and others with *KIR3DL2* (58) (Figure 5). Originally reported as separate genes, *Patr-KIR3DL1/2* and *Patr-KIR3DL3* are now seen as divergent allelic lineages of the same gene, which has been renamed *Patr-KIR3DL1* (140). Human KIR3DL1 binds the Bw4 epitope (58, 74) (Figure 2). The extracellular domains of Patr-KIR3DL1, which are predicted to interact with MHC class I, have considerable similarity to the extracellular domains of KIR3DL1, suggesting that Patr-KIR3DL1 also recognizes Bw4. However, the binding specificity of Patr-KIR3DL1 is different from that of KIR3DL1. It binds some, but not all, Bw4^+^Patr-B, as well as some Patr-B that lack the Bw4 sequence motif (58). In this respect, chimpanzees resemble the rhesus macaque (Old World monkey) by having Bw4 epitopes not recognized by lineage II KIR (141–144). Thus, while homology between ape and human sequences is used to infer the function of ape KIR, it is important to test experimentally the MHC class I specificity of non-human ape KIR.

Chimpanzee lineage III KIR are diverse and numerous, with nine different high-avidity KIR (3, 58, 80, 139, 145) (Figure 5). Eight of these are known to recognize either C1 (three KIR) or C2 (five KIR) (80, 139). Of these eight KIR, five are inhibitory receptors, whereas Patr-KIR3DS6 and Patr-KIR3DS2 are activating KIR with C1- and C2-specificity, respectively. The ninth chimpanzee KIR, Patr-KIR2DS4, is an activating KIR that has a complex specificity, as does its human homolog KIR2DS4 (58, 81). Human KIR2DS4 binds to subsets of C1^+^HLA-C and C2^+^HLA-C as well as certain HLA-A11 allotypes. Patr-KIR2DS4 binds to HLA-A11 and subsets of C1^+^HLA-C but has no affinity for C2^+^HLA-C. In the centromeric region, chimpanzee *KIR* haplotypes exhibit variable combinations of the lineage III *KIR* genes (2–6 genes), which have been generated by the modular shuffling of chromosomal segments (3). Although exhibiting this variability, the chimpanzee *KIR* haplotypes do not form two functional groups, like the human *KIR A* and *KIR B* haplotypes.

None of the chimpanzee lineage III *KIR* genes are true orthologs of human lineage III *KIR* (3). Although *Patr-KIR2DS4* and human *KIR2DS4* have highly homologous sequences, they are not considered true orthologs because they are present at different locations within the *KIR* locus (3). Phylogenetic analysis of the C1- and C2-specific KIR suggests the C1 receptors are ancestral, from which the C2 receptors later evolved (3, 5, 8, 139). Supporting this hypothesis is the presence in orangutans of the C1 ligand and its cognate receptors, but not the C2 ligand and C2-specific KIR (7). The latter are specific to African apes. Differentiating chimpanzees from other African apes is the presence of two types of C2-specific KIR (139). Four chimpanzee C2-specific KIR have methionine at position 44 (M44). The fifth chimpanzee C2-specific KIR, Patr-KIR2DL9, has glutamate at position 44 (E44). E44 confers reactivity with both C1 and C2 (pan-C specificity) to orangutan KIR, contrasting with the C2-specificity of E44 Patr-KIR2DL9 (Figure 5). This difference in specificity is due to the residue at position 45, which is phenylalanine in orangutan KIR and cysteine in Patr-KIR2DL9 (146). KIR that are specific C2 receptors are, therefore, a shared feature of the African hominid KIR, but they independently evolved from C1 receptors on at least two occasions (8, 139).

## Chimpanzee Subspecies Differ in Their MHC Class I and KIR Interactions

The first studies of chimpanzee MHC class I variation focused on *P. t. verus* (*P. t. v*.), the subspecies most highly represented in captive chimpanzee populations (147, 148). A much stronger historical bottleneck distinguishes *P. t. v*., the most western of the subspecies, from the other three subspecies: *P. t. ellioti* (*P. t. e*., Nigeria-Cameroon), *P. t. troglodytes* (*P. t. t*., central), *P. t. schweinfurthii* (*P. t. s*., eastern) (1) (Figure 1). Immunogenetic studies have included the other subspecies by focusing on wild-born chimpanzees resident in African national parks and sanctuaries (12, 100). The current dataset shows clear differences between the subspecies. Maibach et al. (12) targeted the central *P. t. t*. chimpanzee subspecies, which experienced the least population bottleneck. Equivalent *Patr-A, -B*, and *-C* nucleotide diversity was found in the *P. t. t*. and *P. t. v*. subspecies, suggesting that *P. t. v*. chimpanzees selectively regenerated *MHC* diversity following their narrow population bottleneck (12, 93, 149).

The four chimpanzee subspecies cluster in phylogeographically different subgroups: the western-most subspecies, *P. t. v*. and *P. t. e*., comprise one subgroup, and the eastern-most subspecies, *P. t. t*. and *P. t. s*., the other (1, 150) (Figure 1). These relationships suggest that the MHC-KIR interactions of chimpanzee subspecies within a subgroup will be more similar to each other than to subspecies of the other subgroup. Both *P. t. t*. as well as a small number of *P. t. s*. captive chimpanzees have a similarly-reduced frequency of both Bw4^+^ and C1^+^Patr-B compared to *P. t. v*. chimpanzees (Figure 7A). A study of Patr-B in a wild *P. t. s*. chimpanzee population likewise found that the KIR ligands carried by Patr-B were at significantly lower frequency compared to that of a captive population of *P. t. v*. chimpanzees (100). Both *P. t. t*. and *P. t. s*. chimpanzees also exhibit more even frequencies of C1^+^ and C2^+^Patr-C, with a slight skew toward C2, compared to the large C1^+^Patr-C bias of *P. t. v*. chimpanzees (75% C1, 25% C2) (Figure 7A). These observations point to selective differences between the subspecies. The similarity of the two eastern-most subspecies, and their difference from *P. t. v*. chimpanzees, indicates a broader geographical patterning in selection on the interactions between classical MHC class I and KIR. While there are numerous differences between the two phylogeographic subgroups, of note is their variation in disease pressure from SIVcpz. The *P. t. t*. and *P. t. s*. subspecies harbor SIVcpz infection, but the *P. t. v*. and *P. t. e*. subspecies do not (94, 98, 151).

## Lineage III *KIR* Contraction in Bonobos

Despite diverging only ~2 mya (Figure 1), bonobos differ from chimpanzees in having only C2^+^MHC-C (Figure 4). This initial observation, made from studying a limited number of bonobos (6, 137), was confirmed by subsequent analysis of larger bonobo cohorts (11, 12) (Figure 7). Most likely is that bonobos lost C1^+^Papa-C after their divergence from chimpanzees. However, 15% of *Papa-B* alleles encode C1 (Figure 4). *Papa-B* that encode C1 are also maintained in all wild populations, although they accounted for no more than 14% of Papa-B in the populations studied (13). The C1 ligand is similarly represented at low-frequency in the Papa-B of captive populations (11, 12, 137) (Figure 4).

The loss of C1^+^Papa-C is associated with changes in bonobo *KIR*. In the one study of bonobo *KIR*, Rajalingam et al. (4) genotyped a cohort of 11 individuals. Nine of these animals are close-relatives (parent-offspring, full- or half-siblings), which facilitated genomic analysis. In this cohort there is an absence of C1-specific lineage III KIR that corresponds to the absence of C1^+^Papa-C (Figure 5). This suggests that bonobos lost C1 as a ligand for KIR, despite having some C1^+^Papa-B. It is possible that C1^+^Papa-C and the C1-specific lineage III KIR were lost in a bottleneck during bonobo speciation, or, alternatively, a selective sweep that occurred after their divergence from chimpanzees (11–13, 93, 149). Genomic evidence indicates that bonobos did experience a severe bottleneck ~0.5–1 mya years ago, after they diverged from chimpanzees (Figure 1), and again more recently, ~50–100 kya (1). This evidence also shows that there was complex and repeated admixture and gene flow between the ancestors of bonobos and chimpanzees after their divergence, within the last 550,000 years (150). However, MHC class I (-A, -B, and -C) diversity is reduced in bonobos compared to chimpanzees and humans (11–13). That *P. t. v*. chimpanzees experienced a similar population bottleneck to bonobos (1) without comparable loss of C1^+^Patr-C or C1-specific lineage III KIR suggests that selection, rather than bottleneck, drove this loss of Papa-C and KIR diversity in bonobos.

Further evidence for a reduced genetic diversity in bonobos is the contracted bonobo *KIR* haplotype described by Rajalingam et al. (4) (Figure 5). This haplotype contains only three genes, *3DL3, 2DL4*, and *3DLb*. These are *KIR* haplotype framework genes, of lineage *V, I*, and *II*, respectively, so this haplotype is devoid of lineage III *KIR*. Three bonobos are homozygotes for this haplotype, suggesting these three framework *KIR* represent the minimum complement of *KIR* genes that is necessary for survival. This minimal haplotype contrasts with other bonobo *KIR* haplotypes, which resemble some chimpanzee *KIR* haplotypes—which have variable numbers 2–6 of centromeric inhibitory as well as activating lineage III *KIR* situated between the framework *3DL3* and *2DL4* genes. These bonobo *KIR* haplotypes broadly divide between those encoding lineage III KIR that have the capacity to interact with Papa-C and those that do not. This division into two haplotype groups echoes the division of human *KIR* haplotypes into *KIR A* and *KIR B*. Whereas, in bonobos the haplotypic division is based on the ability to interact with Papa-C, in humans the haplotype groups differ by focusing on either inhibitory (*KIR A*) or activating (*KIR B*) KIR. The *3DL3-2DL4* interval is also variable in bonobos, but only two lineage III *KIR* genes have been identified, *Papa-KIR3DL4* and *KIR3DL5*, which are both inhibitory and likely to have C2-specificity conferred by M44 (Figure 5). Both of these bonobo genes have chimpanzee orthologs (3, 4) that have been maintained over the 2 million years since the chimpanzee-bonobo divergence. Nonetheless, the bonobo lineage III *KIR* genes are much reduced, and strictly inhibitory, compared to the more numerous and varied lineage III *KIR* genes of chimpanzees and other great apes.

In contrast to the examples of loss experienced within bonobo *KIR*, bonobos may have moderately expanded the number of their lineage II *KIR* genes. The familial analysis by Rajalingam et al. (4) suggests bonobos likely have two lineage II *KIR* genes: one that can be either *Papa-KIR3DSa*, the only bonobo activating KIR, or inhibitory *Papa-KIR3DLa* (like human KIR3DS1 and KIR3DL1). The other gene is comprised of the *Papa-KIR3DLb* and *c* alleles.

## Human MHC Class I Have Shared Hominid as well as Human-Specific Features

By climbing the evolutionary tree of great apes, we have identified origins of the system of human NK cell education and immune response mediated by the diverse interactions between classical MHC class I and KIR. These comprise some features that are conserved in hominids and others that are human-specific. The multiple *MHC-A* and *MHC-B* genes present in the Asian great apes, the orangutans, have been reduced and functionally refined to single *A* and *B* genes in *Pan* and *Homo*. Gorilla *MHC*s have an intermediate arrangement, in which single, fixed *MHC-A* and *MHC-B* genes, are accompanied by additional genes related to *A* or *B*. The latter are present only on a subset of *MHC* haplotypes and can also be non-functional genes (Figure 4). Such reduction in function appears specifically targeted to genes most related to the *MHC class I* genes of Asian apes—orangutans—than the *MHC class I* genes specific to African apes. During the progressive reduction and refinement in the number and function of African ape MHC-A and MHC-B, the lineage II KIR that recognize MHC-A and -B epitopes remained limited in number (Figure 5). In contrast, lineage III *KIR* genes have increased in number and in their functional specialization with the emergence in hominids of *MHC-C*.

Overall, great ape MHC-A appears dedicated to presenting peptide antigens to CD8 T cells. Bw4, the main ligand for lineage II KIR is poorly represented in great ape MHC-A and A-related molecules (Figure 4). Just two functional Gogo-A or A-related allotypes (06:01 and 05:01) carry the Bw4 ligand. The Bw4 sequence motif is only otherwise encoded in some non-functional *MHC-A*-related genes of orangutan and gorilla (Figure 4). However, 20% of human *HLA-A* alleles encode Bw4 (Figure 4). This unusual feature of HLA-A likely represents a recent emergence of the Bw4 motif within the *HLA-A* gene, one that occurred by gene conversion of an *HLA-A* allele with an allele encoding Bw4^+^HLA-B (152–155). Also distinguishing human HLA-A from MHC-A molecules in other great apes is the A3/11 epitope of HLA-A^*^03 and -A^*^11 allotypes (Figure 4), which is recognized by KIR3DL2, a lineage II KIR molecule (156, 157). At high frequency in modern Asian populations, the HLA-A^*^11 allele was present in archaic Denisovans, who likely passed it on to modern humans through adaptive introgression (110). Thus, the function of MHC-A as a ligand for lineage II KIR appears to be a novel and nearly-unique feature of *Homo*. With the incorporation of these two ligands, the original single framework lineage II *KIR* gene duplicated in humans and the products became specialized receptors for Bw4 (3DL1) and A3/11 (3DL2) (74, 156–158).

Common among hominid MHC-B allotypes are three functional types: one that only presents peptide antigens to CD8 T cell receptors and two that are also ligands for KIR. The latter comprise MHC-B that have a Bw4 epitope, a ligand for lineage II KIR, and MHC-B that have the C1 epitope, a ligand for lineage III KIR (Figure 4). Orangutans are unusual among the hominid species because the three functional types of MHC-B are, to considerable extent, the products of different *MHC-B* genes (Figure 4). The orangutan MHC-B^*^08 gene encodes the C1 epitope recognized by lineage III KIR, the MHC-B^*^03 gene encodes the Bw4 epitope recognized by lineage II KIR, and the other *MHC-B* genes encode allotypes that lack Bw4 or C1 are dedicated ligands for the αβ T cell receptor. Thus, the three functional types originated in the context of different genes, but with subsequent reduction in the number of *MHC-B* genes among the African apes, the different types were ultimately brought together as alleles of a single *MHC-B* gene—a characteristic shared by *Pan* and *Homo* but not *Gorilla* (Figure 4). Distinguishing human HLA-B from the MHC-B of other hominids is an extremely low frequency of C1^+^MHC-B. HLA-B^*^46:01 and HLA-B^*^73:01 are the only C1^+^MHC-B (Figure 4), and both allotypes have geographical distributions and sequences suggesting that modern humans also received them from archaic humans (110). This likely restored C1 within human HLA-B after a previous loss of this epitope.

The C1 epitope originally evolved in the context of an *MHC-B* gene. Subsequently, a C1^+^MHC-B underwent further differentiation to become the *MHC-C* gene (6). This is illustrated by modern orangutans, in which all alleles of the fixed *MHC-C* gene encode C1^+^MHC-C (5, 6, 14). That all ancestral hominid MHC-C allotypes, rather than subsets, carried a C1 ligand set the stage for MHC-C to eventually become the dominant KIR ligand for NK cell education (Figure 4). After *MHC-C* was fixed and the C2 ligand arose from C1 in the African apes, lineage III KIR further diversified and specialized to be receptors specific for either C1 or C2. In gorillas, bonobos, and chimpanzees C2^+^HLA-C allotypes are at higher frequency than C1^+^MHC-C (Figure 4), a dominance that is reflected in the increased number of genes encoding C2-specific lineage III KIR compared to C1-specific lineage III KIR (Figure 5). In striking contrast, a bias of human *HLA-C* alleles encoding the C1 epitope (Figure 4) results in most human populations also having a dominance of C1^+^MHC-C [mean frequency = 0.64 (range: 0.24–0.98)] [calculated from Allele Frequency Net Database, http://www.allelefrequencies.net (126)]. It seems possible that the high frequency of C1^+^MHC-C in humans was a compensatory response to the human-specific loss of C1^+^HLA-B.

## Additional Demands of Human Reproduction Drove *KIR* Haplotype Specialization

A defining feature of the human *KIR* locus was the evolution of two haplotypic forms, *KIR A* and *KIR B* (56, 57, 83). *KIR A* are defined by having mostly inhibitory KIR and limited gene content, particularly that of lineage III *KIR*. *KIR B* have additional activating receptors and are more diverse in their lineage III *KIR* gene content. Both the centromeric and telomeric regions of human *KIR* haplotypes can have either *KIR A* and *KIR B* characteristics (83). Variable telomeric lineage III *KIR*, vs. the variable centromeric lineage III *KIR* found in other great apes, is a feature unique to humans (3, 4, 7, 73, 83, 125, 145). Centromeric *KIR A* and *KIR B* evolved first, with telomeric lineage III *KIR* descending from centromeric genes and then subsequently evolving *KIR A* and *KIR B* characteristics (8, 83). All human populations maintain each form through balancing selection, which suggests that these two forms provide different but essential functions (8, 159–163).

To understand the evolution of these two forms we first considered how MHC-C interactions with lineage III KIR govern processes other than immune responses to cells compromised by infection, cancer and other forms of cellular stress. Interactions of the C1 and C2 epitopes of MHC-C with lineage III KIR also play an essential role reproduction, specifically in embryo implantation and formation of the placenta. This is achieved by interaction of MHC class I (MHC-C, -E, -F, and -G) on fetal extravillous trophoblast cells with their cognate receptors on specialized maternal NK cells that populate the uterine tissue (164). This cooperation leads to the narrow spiral arteries of the uterus being invaded by the trophoblast cells and widened to increase blood supply to the placenta and support fetal development throughout gestation (165). In primates, the extent to which the uterine arteries are invaded varies with the species. Extensive invasion is characteristic of African apes but not of Old World monkeys (166–170). This difference correlates with the emergence in great apes of MHC-C and diversified lineage III KIR (7–10, 14, 73, 121, 125, 128). Orangutan pregnancies have not been studied in depth, but there are indications that the uterus is invaded but to a lesser extent than occurs in African apes (8, 171).

Uterine invasion is carefully coordinated to achieve effective placentation (172). Insufficient invasion of the uterus causes an inadequate blood supply to the placenta and is associated with various pregnancy disorders, including pre-eclampsia, pre-term labor, miscarriage, and stillbirth. Conversely, an over-invasion of the uterus increases the blood supply, leading to large babies and complications in giving birth such as a failure to traverse the birth canal. As MHC-C is the only polymorphic MHC class I expressed by trophoblast, its polymorphism can have major effects on these outcomes of pregnancy (86, 87, 172). Unique to the biology of uterine cells is that they physiologically interact with one MHC-C allotype of maternal origin and a second of paternal origin, both of which are expressed by the extravillous trophoblast cells (87). Reproductive outcomes correlate with the MHC-C type of the mother, the paternal MHC-type inherited by the fetus, and the *KIR* haplotypes of the mother (86, 87, 172, 173). However, the same combined type that is disadvantageous in reproductive contexts can be advantageous against disease, and vice versa. Such is the case for females who are homozygous for C1^+^HLA-C and KIR A (86, 87, 173). They are at increased risk of pre-eclampsia if they become pregnant with heterozygous embryos that carry paternal C2^+^HLA-C. This may be because their uterine C1^+^HLA-C-educated NK cells (via C1-specific KIR2DL3) respond to the reduced amount of C1^+^HLA-C on the heterozygous embryo as missing self. Additionally, or alternatively, the inhibitory C2 receptor found on the KIR A haplotype, KIR2DL1, would inhibit uterine NK cells upon recognition of the fetal C2^+^HLA-C, also potentially resulting in poor placentation. However, when faced with hepatitis C virus, this same compound *MHC-KIR* genotype is associated with more positive outcomes of infection (85, 174). Tradeoffs such as these suggest that the dual roles for MHC-C in immunity and reproduction have likely contributed to the evolution and balanced maintenance of the two haplotypic forms of KIR under varying selection from disease and reproduction during the evolution of *Homo*. It is hypothesized that such compromises in humans, but not other apes, were driven by the energetic and reproductive demands that facilitated the growth of the disproportionately large brains of human babies (8, 163, 172).

This balance is critical to maintain and is struck in population-specific patterns of co-evolution, particularly when unbalanced distributions could decrease fitness. Populations with high C2^+^HLA-C would be at particular risk for pregnancy disorders associated with poor placentation. The KhoeSan, among other African populations, carry a high frequency of C2^+^HLA-C (63%) (175). However, among the ten alleles found for KIR2DL1 within the KhoeSan are two unusual alleles, KIR2DL1^*^022 and ^*^026, whose characteristics reduce such risk (23). These alleles switched, by point mutations in parent alleles, from risk-associated inhibitory C2 receptors to an inhibitory C1 receptor (^*^022) and a molecule that is deficient in signaling (^*^026), thereby resulting in lack of NK cell education. Both mutated alleles are also found at higher frequencies than the alleles from which they descended, suggesting they have been positively selected, likely due to their protective effects against poor-placentation.

Tipped in the other direction are the Yucpa, an indigenous population living along the border of Venezuela and Colombia. They have a high-frequency of the C1^+^C^*^07:02 (176), an allele thought to be introgressed into modern humans via admixture with Neandertals in Asia and then brought to the Americas by migrating humans (110). C^*^07:02, alone, has a frequency of 76% in the Yucpa. Combined with the other C1^+^HLA-C-bearing allotypes, C1^+^HLA-C has a frequency of 82% (176). Amidst this high frequency of C1^+^HLA-C, the Yucpa *KIR A* and *B* haplotype frequencies are balanced (46% *KIR A*, 54% *KIR B*), but there have been changes to the C1 inhibitory receptor, KIR2DL3, carried by *KIR A* haplotypes. The Yucpa population has three KIR2DL3 allotypes, the parental Eurasian 2DL3^*^001 and two descendants. Mutations have rendered the descendants less effective as C1 receptors: one is completely ineffective as a null allele (^*^008N), and the other has been substantially weakened in its avidity for C1 (^*^009). These two mutant forms account for 91% of *KIR A* haplotypes, thus attenuating the interactions between C1 and its receptor. However, this modification is *KIR A* specific, as *KIR B* haplotypes still encode high-avidity inhibitory receptors for C1 as 2DL3^*^001 or 2DL2^*^003.

As MHC class I ligand and KIR interactions are important to both survival and reproduction it is, therefore, not surprising that the essential functional elements, the different ligand types and their cognate receptors, are maintained between species and populations. Throughout hominid evolution maintaining Bw4-lineage II KIR and C1-lineage III KIR interactions for NK cell functioning has likely been critical to survival. Even populations known to have suffered severe bottleneck and exhibiting reduced MHC and KIR diversity, such as the indigenous South American Yucpa (176), maintain these core MHC class I ligand and KIR interactions. However, the apparent loss of the C1-lineage III KIR arm of NK cell immunity in bonobos suggests that populations can, at least, survive the loss of this arm (11–13, 137) (Figure 4). C2-lineage III KIR interactions appear necessary for survival as they are maintained among both bonobos and the Yucpa. Because C2-specific NK cell education can only be achieved through MHC-C there has likely been strong selection to maintain C2-specific interactions since the emergence of C2 within the African ape lineage. As no such African hominid species or population has yet been described to completely lack Bw4 or C2 and their cognate KIR, it is likely that any population that experienced such a loss has not survived. Appearing unnecessary for survival, however, is the human-specific A3/11-lineage II KIR mode of NK cell education, which is absent among the Yucpa (176). This likely reflects the shorter evolutionary history of A3/11 within modern humans, having been recently introgressed through admixture with archaic humans (110).

## Summary

The overall pattern of MHC-KIR evolution in the apes is one of refinement and reduction of MHC class I accompanied by expansion and refinement of KIR. The elimination, reduction, and functional refinement of the orangutan-like *MHC-A* and *-B* in the African apes is associated with minor, yet significant, changes in the number of lineage II *KIR* genes. During hominid evolution MHC-A has mostly been a dedicated ligand for T cells, but after the *Homo* lineage diverged from that of *Pan*, HLA-A emerged to also serve as ligand for lineage II KIR with a gene conversion of Bw4 from MHC-B and archaic introgression of the A11 epitope. Acquisition of this novel function was associated with the emergence of two separate lineage II *KIR* genes that are specialized receptors for each of these ligands. By contrast, MHC-B has been a consistent source of ligands for both lineage II and III KIR throughout hominid evolution, maintaining a portion of allotypes encoding their Bw4 and C1 ligands, respectively. Initially, encoding of these ligands was segregated between multiple *MHC-B* genes. As the number of *MHC-B* genes was reduced, KIR ligand encoding converged within a single *MHC-B*.

**Figure 8:**
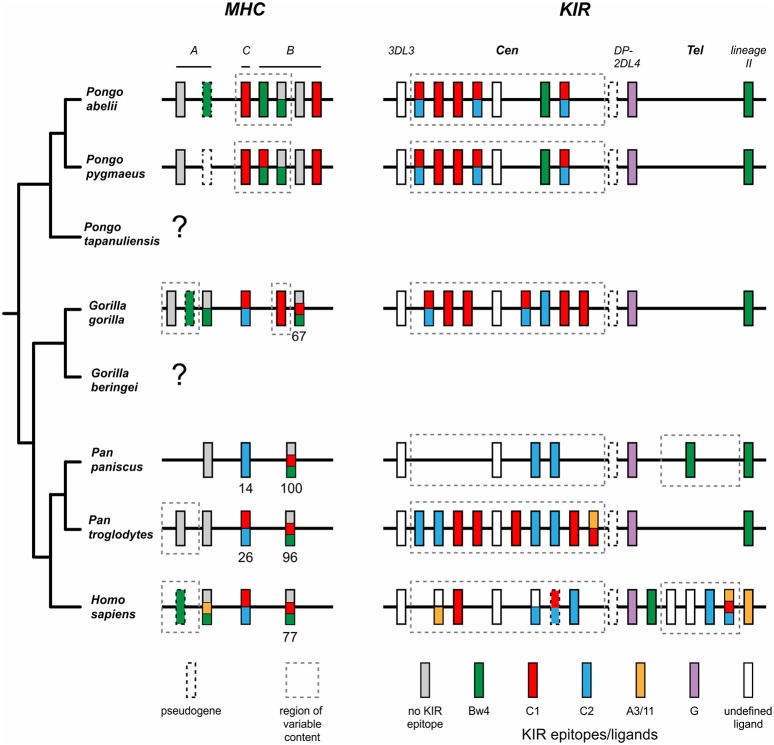
Summary of *MHC* and *KIR* gene content in great apes. The cladogram on the left shows relationships among the great apes. Under *MHC* is a schematic representation of the gene content for the *MHC-A, -B*, and *-C* regions. A gene, or genes, enclosed by a dotted rectangle is in a region of variable gene content. A dashed rectangle indicates a pseudogene. Color-coding within the rectangle representing a gene shows the possible KIR ligands encoded by that *MHC* gene. This color-coding is not proportional to the frequency of the encoded ligand. Numbers beneath *MHC-B* and *-C* in gorillas, chimpanzees, and humans give the frequency of −21T in the allotypes of each gene. No number denotes a frequency of 100% for −21M. On the right under *KIR* is a schematic representation of the *KIR* locus gene content in each species. The framework genes (*3DL3, DP-2DL4, lineage II*) are indicated. As for *MHC, KIR* pseudogenes are indicated by a dashed rectangle and regions of variable gene content are surrounded by a dotted line. The ligand-specificities encoded by each *KIR* gene are indicated by the same color-coding used for *MHC*.

In contrast to the reduction and refinement seen for *MHC-A* and -*B*, the *MHC-C* gene, which emerged as a variable gene on the haplotypes of the common ancestor of the great apes, became fixed in the common ancestor of the African apes (Figure 8). The emergence of *MHC-C* partnered with a rapid expansion of lineage III *KIR* genes. Highlighting this fact, few lineage III *KIR* orthologs are found except in species of the same genus that recently diverged (Figures 1, 8). Initial hominid MHC-C and lineage III KIR interactions were mediated entirely by the C1 ligand. *MHC-C* fixation and the emergence of its C2 ligand in the common ancestor of African ape drove the further expansion of lineage III *KIR* and their specialization for either ligand. Further refinement and specialization of the human *KIR* locus resulted in *KIR A* and *KIR B* centromeric and telomeric *KIR* haplotypes, differing primarily in lineage III *KIR*, that are variably associated with disease and reproductive outcomes. Distinctions within the diverse great ape species and subspecies, such as the unique contraction observed within the bonobo of the *MHC* and *KIR* loci and the ligand differences between chimpanzee subspecies, present new opportunities to better understand how differences in selection contribute to the shaping of MHC and KIR diversity in both human and non-human great apes.

The dynamic changes within the classical MHC class I and KIR ligand-receptor system over the course of hominid evolution contrast the relative stability of the non-classical MHC-E and CD94:NKG2 system of NK cell education. However, the −21T polymorphism of the MHC-B leader sequences, which biases individuals toward KIR-mediated education of NK cells, critically emerged among the African ape lineage alongside the fixation of *MHC-C* and further expansion and diversification of lineage III *KIR* (Figure 8). Essential to the co-evolution of two systems was concomitant change to MHC-B. MHC-B has increased expression compared to MHC-A or MHC-C. Elimination of additional *MHC-B* gene copies likely made changes to cell surface MHC-E based on MHC-B position −21 polymorphism more pronounced, with more surface MHC-E driven by −21M bolstering the education of CD94:NKG2A NK cells, and reduced surface MHC-E associated with −21T favoring the education of NK cells via KIR. Under apparent mutual selection for polymorphism favoring the more novel and dynamic KIR system, both systems of NK cell education and immune responses evolved independently yet in concert with the other during hominid evolution. Together these data have shed new light on the evolutionary dance between two cooperating systems of MHC class I and NK cell receptors.

## Author Contributions

All authors listed have made a substantial, direct and intellectual contribution to the work, and approved it for publication.

### Conflict of Interest Statement

The authors declare that the research was conducted in the absence of any commercial or financial relationships that could be construed as a potential conflict of interest.
